# Associations of exercise frequency, exercise session duration, and sleep duration with depressive symptoms among Chinese adolescents: a complex-survey cross-sectional analysis of CFPS2022

**DOI:** 10.3389/fspor.2026.1924903

**Published:** 2026-09-07

**Authors:** Mingyuan Li, Bingyang Zhai

**Affiliations:** 1 Department of Military and Physical Education, Jiangxi Institute of Fashion Technology, Nanchang, China; 2 Department of Sports Science, Kunsan National University, Kunsan, Republic of Korea

**Keywords:** adolescents, CFPS, complex survey, depressive symptoms, exercise frequency, physical exercise, sleep duration

## Abstract

**Background:**

Exercise and sleep are modifiable behaviors related to adolescent mental health, but the relevance of specific exercise-frequency categories, participant session duration, and nonlinear sleep duration remains uncertain.

**Methods:**

We analyzed 2,861 adolescents aged 10–19 years from the 2022 China Family Panel Studies. Survey-weighted regression incorporated individual weights, strata, and primary sampling units. Exercise frequency, any exercise participation, participant session duration, and spline-modeled sleep duration were examined in separate models with continuous eight-item Center for Epidemiologic Studies Depression Scale (CES-D8) scores as the primary outcome.

**Results:**

CES-D8 scores differed across exercise-frequency categories (*p* = 0.002), but the pattern was not monotonic. The largest adjusted difference was observed for once-daily exercise vs. never exercising (*B* = −1.08; 95% CI, −1.62 to −0.53). Any exercise participation was associated with a modestly lower score (*B* = −0.57; 95% CI, −1.01 to −0.12), whereas session duration among 2,167 exercise participants was not associated with CES-D8 scores (*B* per 30 min = 0.004; 95% CI, −0.16–0.17). Sleep duration was nonlinearly associated with CES-D8 scores (overall *p* < 0.001; nonlinearity *p* = 0.004); relative to 8 h, scores were higher at 6 h (*B* = 1.03; 95% CI, 0.55–1.51) and 7 h (*B* = 0.53; 95% CI, 0.30–0.76). Sensitivity analyses were broadly consistent, and multiple-imputation estimates for exercise participation, exercise-frequency contrasts, and sleep-duration contrasts were similar to the complete-case estimates.

**Conclusion:**

Exercise frequency and sleep duration were modestly associated with depressive-symptom scores, whereas participant session duration showed no clear association. The cross-sectional findings do not establish causality or support a specific exercise prescription.

## 1 Introduction

Adolescent depressive symptoms have become an important concern in developmental psychology and public health. Adolescence is a formative developmental period marked by substantial emotional, cognitive, and social changes, during which health-related behavioral patterns begin to take shape (1). Depressive symptoms during adolescence have been associated with poorer academic performance and social functioning, as well as adverse psychosocial outcomes that may persist into adulthood (2, 3). Existing evidence indicates that elevated depressive symptoms are prevalent among adolescents, although estimates vary considerably across populations and assessment instruments (4, 5). Identifying modifiable behaviors associated with depressive symptoms is therefore important for both adolescent health research and population-level prevention.

Physical activity is a modifiable health behavior associated with adolescents’ physical and mental well-being (6). Observational studies have generally reported inverse associations between physical activity or sport participation and depressive symptoms among adolescents (7, 8), while trials of structured exercise programs have reported reductions in depressive-symptom scores (9). Potential explanations include psychosocial pathways such as self-efficacy and self-esteem, while social and neurobiological pathways have also been proposed; however, evidence for specific mechanisms remains limited (10). Moreover, exercise is a multidimensional behavior that varies in frequency, session duration, intensity, modality, and context (11).

Studies of Chinese adolescents have likewise reported inverse associations between physical activity and depressive symptoms. For example, one large cross-sectional study found that moderate and high levels of physical activity were associated with fewer depressive symptoms, while a more recent school-based study reported nonlinear associations of weekly activity frequency and total weekly activity time with depressive-symptom scores (12, 13). However, some previous studies have operationalized physical activity as a continuous measure of weekly exercise time or as a composite questionnaire score, and continuous duration measures have sometimes assigned a value of zero to nonparticipants. These approaches may obscure distinctions among exercise participation, frequency, and session duration (14, 15). Treating ordered exercise-frequency categories as a single numeric score may obscure category-specific patterns and implicitly impose a linear trend across frequency levels (16). Moreover, session duration is undefined among adolescents who report no exercise; coding nonparticipants as having zero-minute sessions conflates two distinct constructs—exercise participation and session duration (17). It is therefore necessary to distinguish any exercise participation and specific frequency categories from variation in session duration among exercise participants.

Sleep duration is another modifiable health behavior closely associated with adolescent mental health. Sleep is closely related to learning, attention, cognition, and emotional processing. Experimental sleep restriction has been shown to increase fatigue and irritability and impair emotion regulation, while shorter sleep duration and poorer sleep quality have also been associated with greater depressive symptoms during adolescence (18–20). Longitudinal studies suggest that sleep problems and depressive symptoms may predict one another over time during adolescence (21). Physical activity, sedentary screen time, and sleep are interrelated components of adolescents’ daily routines, and day-to-day studies have identified temporal associations among these behaviors (22). School start-time changes have also been associated with changes in sleep duration, physical activity, and screen time (23). Although physical activity, sedentary behavior, and sleep occur within a finite 24-hour period and may therefore be behaviorally interdependent, the present study did not directly assess time-use substitution or determine whether exercise displaced sleep or other daily activities (24).

Despite this evidence, several methodological questions remain. First, modeling exercise frequency as a single ordered score may impose assumptions of equal spacing and monotonic change across categories, whereas retaining the original response categories allows category-specific differences to be estimated (25). Second, exercise participation and the amount of exercise among participants represent distinct dimensions and may warrant separate analyses (26). Third, the association between sleep duration and depressive symptoms may be nonlinear and asymmetric; models based only on a linear term or broad sleep categories may therefore fail to capture differences between shorter and longer sleep durations (27). Finally, because the CES-D includes a sleep-related item, sensitivity analyses excluding this item can help assess possible content overlap when sleep is examined as an exposure (28). Accordingly, the present study examined the original exercise-frequency categories, any exercise participation, participant-only session duration, nonlinear sleep associations, and sleep-item overlap within a complex-survey analysis of Chinese adolescents.

Using data from adolescents aged 10–19 years in the 2022 China Family Panel Studies, the present study addressed four related questions: (1) whether depressive-symptom scores differed across the original eight exercise-frequency categories without imposing a monotonic dose–response assumption; (2) whether any exercise participation was associated with depressive symptoms in the full adolescent sample; (3) whether usual session duration was associated with depressive symptoms among exercise participants after adjustment for exercise frequency; and (4) whether sleep duration showed overall and nonlinear associations with depressive symptoms when modeled flexibly.

## 2 Materials and methods

### 2.1 Study design and participants

CFPS is a longitudinal household survey conducted by the Institute of Social Science Survey, Peking University. This cross-sectional analysis used the 2022 wave. Individual self-reports were linked to household income and household size using the 2022 household identifier. All outcome and behavioral variables were obtained from adolescents’ Individual Self-report Questionnaires; proxy responses were not used to replace missing self-reported values. Of 27,001 respondents, 3,259 were aged 10–19 years. After excluding respondents with missing or invalid outcome, exposure, covariate, household, or survey-design information, the fixed complete-case sample included 2,861 adolescents from 6 strata and 166 primary sampling units (PSUs). This sample was used for the primary and non-imputation sensitivity analyses; a broader sample was used for multiple imputation. Detailed construction of the fixed complete-case sample is provided in Supplementary Table S1A.

All survey-weighted descriptive statistics and regression models incorporated the CFPS2022 individual cross-sectional weight, stratum, and PSU identifiers. The CFPS was approved by the Peking University Biomedical Institutional Review Board (IRB00001052-14010), and informed consent was obtained by the original survey team. The present study used de-identified secondary data and involved no additional participant contact.

### 2.2 Measures

#### 2.2.1 Depressive symptoms

The CES-D8 assessed symptoms during the previous week. Eight items were scored from 1 to 4, with 2 positive items reverse-coded and summed to a total score of 8–32; higher scores indicated more severe symptoms. Continuous CES-D8 was the primary outcome. A raw score of 16 or higher was used only as an operational binary sensitivity outcome. For a sensitivity analysis, the restless-sleep item was excluded, yielding a CES-D7 score ranging from 7 to 28. This score was not treated as an independently validated scale, and no cutoff was applied.

#### 2.2.2 Exercise

Exercise frequency referred to physical fitness or leisure activities during the previous 12 months. In the original CFPS questionnaire, response codes 1–7 represented progressively higher exercise-frequency categories, whereas code 8 indicated never exercising. The response categories were: 1 = less than once per month; 2 = more than once per month but less than once per week; 3 = 1–2 times per week; 4 = 3–4 times per week; 5 = more than 5 times per week; 6 = once per day; 7 = at least twice per day; and 8 = never. For analysis, the categories were reordered and entered as an eight-level categorical factor, with never exercising as the reference category. The numerical codes were used only to identify the response categories and were not treated as an equally spaced continuous scale. Any exercise participation was examined separately in the full sample. Usual session duration was analyzed per 30 min among exercise participants only; never-exercisers were not assigned 0 min.

#### 2.2.3 Sleep and covariates

Usual daily sleep duration was recorded in hours. Primary models adjusted for age, gender, urban-rural residence, log-transformed household income per capita, and household size. Educational years, self-rated health, change in health, and happiness were added only in sensitivity analyses because their temporal and causal positions were uncertain.

Other potentially relevant factors, including screen use, study time, chronic health conditions, family structure, parental mental health, and school characteristics, were not included because harmonized full-sample measures were unavailable or conditionally observed.

### 2.3 Statistical analysis

Sample characteristics were summarized using unweighted counts and survey-weighted means, standard deviations, proportions, and 95% confidence intervals. Standard errors, confidence intervals, and Wald tests were estimated by Taylor-series linearization.

Survey-weighted linear regression modeled continuous CES-D8 scores. Exercise frequency, any exercise participation, participant session duration, and sleep duration were evaluated in separate exposure-specific models. For the exploratory age-group interaction assessment, participant session duration was represented using a spline to allow for possible nonlinearity. Exercise frequency was entered as an eight-category factor, with never exercising as the reference. The overall association was assessed using a survey-adjusted joint Wald test, and category-specific adjusted mean differences were estimated without assuming equal spacing or a monotonic linear trend across categories. The participant session-duration model additionally adjusted for exercise-frequency category.

Sleep duration was modeled using a natural cubic spline with boundary knots at 6 and 10 h and internal knots at 8 and 9 h, corresponding to survey-weighted percentiles of the observed distribution. Eight hours was the reference; contrasts were estimated at 6, 7, 9, and 10 h. Overall and nonlinear components were tested jointly. Category-specific exercise-frequency contrasts and prespecified sleep contrasts were not adjusted for multiple comparisons and were interpreted with the corresponding omnibus tests, effect sizes, confidence intervals, and sensitivity analyses.

Sensitivity analyses used continuous CES-D7, the operational binary CES-D8 outcome, and alternative covariate sets. The binary outcome was analyzed using survey-weighted logistic regression and reported as odds ratios. Included and excluded age-eligible respondents were compared descriptively using available data and absolute standardized mean differences (Supplementary Table S1B).

Multiple imputation by chained equations used 20 datasets and 20 iterations in a broader model-specific sample of 3,019 adolescents (Supplementary Table S1C). Of 158 respondents not included in the fixed complete-case sample, 155 had at least 1 eligible variable imputed and 3 were complete on all variables used in the imputation models but had invalid participant session-duration values; the latter were retained because session duration was neither imputed nor included in the reported imputation models. Single-parameter coefficients and prespecified contrasts were combined using Rubin rules; multiparameter pooled omnibus tests were not reported.

Exploratory interactions with age group (10–14 vs. 15–19 years) were assessed using survey-adjusted joint Wald tests. Selected model checks included collinearity, leave-one-PSU-out stability, and PSU-clustered cross-validation assessments. Analyses were conducted in R using 2-sided tests; interpretation emphasized effect magnitude, precision, and consistency rather than a significance threshold alone.

## 3 Results

### 3.1 Sample characteristics

The fixed complete-case sample included 2,861 adolescents: 1,557 aged 10–14 years and 1,304 aged 15–19 years. A total of 2,167 reported exercising and 694 reported never exercising. Mean CES-D8 was 12.20 (SD, 3.54), mean sleep duration was 8.27 h/day (SD, 1.36), and 473 adolescents met the operational CES-D8 threshold, corresponding to a weighted proportion of 16.45% (95% CI, 14.53%–18.56%) (Table 1).

**Table 1:** Survey-weighted characteristics of the CFPS2022 adolescent analytic sample (*N* = 2,861).

Characteristic	No.	Survey-weighted estimate
Demographic characteristics		
Age, mean (SD), y	2,861	14.22 (2.92); 95% CI, 14.06–14.38
Age 10–14 y	1,557	54.26% (95% CI, 51.67%–56.85%)
Age 15–19 y	1,304	45.74% (95% CI, 43.15%–48.33%)
Female	1,319	46.45% (95% CI, 43.89%–49.00%)
Urban residence	1,276	52.89% (95% CI, 47.43%–58.36%)
Household income per capita, median (IQR), RMB	2,861	19,000 (11,250–30,333)
Household size, mean (SD)	2,861	5.03 (1.88); 95% CI, 4.87–5.20
Depressive symptoms and sleep		
CES-D8 score, mean (SD)	2,861	12.20 (3.54); 95% CI, 11.98–12.42
Raw CES-D8 ≥ 16	473	16.45% (95% CI, 14.53%–18.56%)
Sleep duration, mean (SD), h/d	2,861	8.27 (1.36); 95% CI, 8.21–8.34
Exercise		
Any exercise participation	2,167	76.95% (95% CI, 74.13%–79.76%)
Never exercises	694	23.05% (95% CI, 20.24%–25.87%)
Less than once per month	54	1.76% (95% CI, 1.23%–2.29%)
More than once per month but less than once per week	191	7.05% (95% CI, 5.59%–8.51%)
1–2 times per week	719	25.02% (95% CI, 22.94%–27.10%)
3–4 times per week	432	14.59% (95% CI, 12.63%–16.55%)
More than 5 times per week	139	5.14% (95% CI, 4.03%–6.26%)
Once per day	473	16.85% (95% CI, 14.93%–18.76%)
At least twice per day	159	6.54% (95% CI, 4.96%–8.12%)
Session duration, mean (SD), min; participants only	2,167	52.96 (43.69); 95% CI, 49.74–56.18

Counts are unweighted; all other estimates are survey-weighted using the CFPS2022 individual cross-sectional weight, 6 strata, and 166 PSUs. Household income was modeled as log(income per capita + 1). CES-D8 ≥ 16 was used only as an operational sensitivity outcome. Session duration was defined only for exercise participants.

### 3.2 Exercise and depressive symptoms

CES-D8 scores differed across exercise-frequency categories after adjustment, *F*(7,148) = 3.54, *p* = 0.002 (Table 2). Relative to never exercising, the largest difference was observed for once-daily exercise (*B* = −1.08; 95% CI, −1.62 to −0.53), whereas the estimate for at least twice-daily exercise was close to 0 (*B* = −0.10; 95% CI, −1.05–0.85). The category pattern was not monotonic.

**Table 2:** Survey-weighted associations of exercise and sleep duration with continuous CES-D8 scores.

Analysis or contrast	N	*B* or *F*	95% CI	*p* value
Exercise-frequency categories (reference: never)				
Overall association	2,861	*F*(7,148) = 3.54		.002
Less than once per month	2,861	−0.273	−1.441 to 0.895	.645
More than once per month but less than once per week	2,861	0.315	−0.577 to 1.206	.486
1–2 times per week	2,861	−0.508	−1.055 to 0.038	.068
3–4 times per week	2,861	−0.711	−1.322 to −0.099	.023
More than 5 times per week	2,861	−0.752	−1.399 to −0.106	.023
Once per day	2,861	−1.076	−1.617 to −0.534	<0.001
At least twice per day	2,861	−0.103	−1.054 to 0.848	.831
Exercise participation and session duration				
Any exercise participation vs. never	2,861	−0.568	−1.013 to −0.123	.013
Session duration per 30 min; participants only; frequency-adjusted	2,167	0.004	−0.161 to 0.170	.958
Sleep-duration spline (reference: 8 h)				
Overall association	2,861	*F*(3,152) = 7.16		<0.001
Departure from linearity	2,861	*F*(2,152) = 5.66		.004
6 h vs. 8 h	2,861	1.031	0.549 to 1.512	<0.001
7 h vs. 8 h	2,861	0.530	0.297 to 0.764	<0.001
9 h vs. 8 h	2,861	−0.420	−0.834 to −0.007	.047
10 h vs. 8 h	2,861	−0.233	−0.668 to 0.202	.292

B values are unstandardized adjusted mean differences in CES-D8 points. Exposure domains were evaluated in separate models adjusted for age, gender, urban-rural residence, household income per capita, and household size. The session-duration model additionally adjusted for exercise-frequency category. Category-specific exercise and sleep contrasts were not adjusted for multiple comparisons.

Any exercise participation was associated with a modestly lower CES-D8 score (*B* = −0.57; 95% CI, −1.01 to −0.12; *p* = 0.013). Among exercise participants, session duration was not associated with CES-D8 after adjustment for frequency and primary covariates (*B* per 30 min = 0.004; 95% CI, −0.16–0.17; *p* = 0.958) (Table 2).

### 3.3 Sleep duration

Sleep duration was associated with CES-D8 scores overall, *F*(3,152) = 7.16, *p* < 0.001, with evidence of nonlinearity, *F*(2,152) = 5.66, *p* = 0.004 (Table 2). Relative to 8 h, adjusted scores were higher at 6 h (*B* = 1.03; 95% CI, 0.55–1.51) and 7 h (*B* = 0.53; 95% CI, 0.30–0.76), modestly lower at 9 h (*B* = −0.42; 95% CI, −0.83 to −0.01), and not clearly different at 10 h (*B* = −0.23; 95% CI, −0.67–0.20).

### 3.4 Sensitivity analyses

The overall patterns were broadly similar when depressive symptoms were represented using CES-D7 or the operational binary CES-D8 outcome (Table 3). The overall sleep-duration association remained evident across covariate specifications, although evidence of nonlinearity weakened in some expanded models (Supplementary Table S2A). Multiple-imputation estimates for exercise participation, exercise-frequency contrasts, and the prespecified sleep-duration contrasts were broadly consistent with the complete-case estimates; the 9-hour vs. 8-hour sleep contrast was less precise after imputation (Supplementary Table S2B). No statistically detectable age-group interactions were observed, and diagnostics did not identify an influential single PSU or problematic collinearity in the primary models (Supplementary Table S2C).

**Table 3:** Sensitivity analyses using alternative depressive-symptom outcomes.

Outcome	Analysis	N	Estimate (95% CI) or *F*	*p* value
Continuous CES-D7				
CES-D7	Exercise frequency: overall association	2,861	*F*(7,148) = 3.55	.001
CES-D7	Any exercise participation vs. never	2,861	−0.483 (−0.868 to −0.099)	.014
CES-D7	Session duration per 30 min; participants only	2,167	0.015 (−0.127 to 0.156)	.840
CES-D7	Sleep duration: overall spline association	2,861	*F*(3,152) = 5.84	<0.001
CES-D7	Sleep duration: departure from linearity	2,861	*F*(2,152) = 4.77	.010
Operational binary CES-D8 (raw score ≥16)				
CES-D8 ≥ 16	Exercise frequency: overall association	2,861	*F*(7,148) = 2.39	.024
CES-D8 ≥ 16	Any exercise participation vs. never	2,861	OR, 0.644 (0.470–0.881)	.006
CES-D8 ≥ 16	Session duration per 30 min; participants only	2,167	OR, 0.989 (0.861–1.137)	.880
CES-D8 ≥ 16	Sleep duration: overall spline association	2,861	*F*(3,152) = 3.25	.023
CES-D8 ≥ 16	Sleep duration: departure from linearity	2,861	*F*(2,152) = 4.14	.018

CES-D7 excluded the restless-sleep item. The binary CES-D8 outcome was operational and was not interpreted as a clinical diagnosis. Session-duration analyses were restricted to exercise participants and adjusted for exercise frequency. OR, indicates odds ratio.

## 4 Discussion

Using data from CFPS2022, this study examined the associations of exercise participation, exercise-frequency categories, participant session duration, and sleep duration with depressive symptoms among Chinese adolescents. Four main findings emerged. First, depressive-symptom scores differed across the original exercise-frequency categories, but the pattern was not monotonic. Second, adolescents reporting any exercise had modestly lower CES-D8 scores than those who never exercised. Third, usual session duration was not associated with depressive symptoms among exercise participants after accounting for exercise frequency. Fourth, sleep duration showed a nonlinear association with depressive symptoms, with the clearest differences occurring at shorter sleep durations. Results were generally similar under the alternative depressive-symptom definitions. In the multiple-imputation analyses, estimates for exercise participation, exercise-frequency categories, and the prespecified sleep-duration contrasts were broadly consistent with the complete-case results, although the 9-hour vs. 8-hour sleep contrast was less precise. Exercise estimates were attenuated after additional covariate adjustment.

### 4.1 Exercise participation, exercise frequency, and depressive symptoms

The inverse association between any exercise participation and depressive symptoms is broadly consistent with observational and review evidence linking physical activity with fewer depressive symptoms and better mental health among adolescents (29, 30). Potential psychosocial pathways include improved self-perceptions, perceived competence, and opportunities for social connection, although evidence identifying specific mechanisms remains incomplete (31, 32). Adolescents who exercise may also differ from those who never exercise in health status, social resources, school engagement, and other characteristics that were not fully captured in the present data. The observed association should therefore be interpreted as a modest adjusted group-level association rather than evidence that exercise participation directly reduces depressive symptoms.

Retaining the original exercise-frequency categories provided a more differentiated picture than treating frequency as a continuous or ordered dose. The largest adjusted difference was observed for once-daily exercise, whereas the estimate for exercising at least twice daily was close to zero. The findings therefore did not support a simple interpretation that progressively more frequent exercise was associated with progressively fewer depressive symptoms.

Several measurement and contextual features may be relevant to this nonmonotonic pattern. The frequency item did not capture exercise intensity, activity type, competitive level, motivation, timing, or social context. Consequently, it was not possible to determine whether the once-daily and highest-frequency categories represented comparable forms or contexts of exercise. Previous research suggests that associations with depressive symptoms may vary across physical activity contexts, while intensive youth sport may involve substantial training, performance, and academic demands (33, 34). Estimates for the extreme-frequency categories were also less precise because relatively few adolescents were represented in these groups. Finally, longitudinal evidence suggests that physical activity and depressive symptoms may be bidirectionally associated; depressive symptoms may therefore also be associated with lower willingness or ability to exercise (35).

The magnitude of the observed differences should also be considered. Any exercise participation corresponded to approximately 0.57 fewer CES-D8 points, and the largest frequency-category estimate was approximately 1.08 points on a scale ranging from 8 to 32. A minimally important difference has not been established for the CES-D8 in this population. Accordingly, these estimates indicate modest group-level associations and do not establish that once-daily exercise is optimal or that exercising at least twice daily is ineffective. These findings illustrate the value of retaining the original frequency categories when the shape of the association is uncertain, while providing no basis for recommending a specific exercise frequency.

### 4.2 Participant session duration and depressive symptoms

Session duration was not associated with depressive symptoms among adolescents who reported exercising. This distinction is important because participation, frequency, and session duration capture different aspects of exercise behavior. The findings indicate that usual session duration was not associated with additional variation in depressive-symptom scores after adjustment for exercise frequency and the primary covariates.

The null estimate should not be interpreted as evidence that the amount or qualitative characteristics of exercise are irrelevant to adolescent mental health. Session duration alone does not measure total weekly exercise volume and cannot distinguish moderate from vigorous activity, individual from group exercise, compulsory from voluntary participation, or enjoyable from stressful activity. Two adolescents reporting the same session duration may therefore have very different exercise exposures. Review evidence further suggests that depressive-symptom outcomes may vary across exercise modalities and intervention characteristics, including session duration and weekly frequency, rather than showing a consistent association with session duration alone (36).

Methodologically, restricting the duration analysis to exercise participants avoided assigning zero-minute sessions to adolescents who never exercised. Such coding would conflate two distinct questions: whether an adolescent participated in exercise and, among participants, how long a usual session lasted. In the present study, exercise participation and frequency were associated with depressive symptoms, whereas participant-only session duration was not. Future studies incorporating device-measured intensity and total weekly exercise volume are needed before firmer conclusions can be drawn about the amount of exercise relevant to adolescent depressive symptoms.

### 4.3 Sleep duration and depressive symptoms

Sleep duration was nonlinearly associated with CES-D8 scores, with the most consistent differences observed at shorter durations. Compared with adolescents reporting 8 h of sleep, those reporting 6 or 7 h had higher depressive-symptom scores. This pattern is consistent with experimental evidence showing that sleep restriction can adversely affect adolescents’ mood, subjective alertness, cognitive performance, and emotional functioning (37, 38). Greater sleepiness and difficulties in emotional or executive functioning may be relevant explanations, although these mechanisms were not measured in the present study.

The results did not support a clear, symmetric U-shaped association. Although the estimate for 9 h was modestly lower than the estimate for 8 h in the complete-case analysis, the difference was slightly attenuated and its confidence interval included zero after multiple imputation. The estimate for 10 h was also imprecise and did not clearly differ from the reference. Accordingly, the principal sleep-related finding was higher depressive-symptom scores at shorter durations; the results did not establish 9 h as an optimal duration or suggest that longer sleep was uniformly protective.

The CES-D7 sensitivity analysis, which removed the restless-sleep item from the depressive-symptom score, produced a similar overall pattern. This suggests that the association was not explained entirely by direct content overlap between reported sleep duration and the sleep-related CES-D8 item. However, the CES-D7 was constructed specifically for sensitivity analysis and has not been independently validated as a separate instrument. It should therefore be interpreted as a check on measurement overlap rather than as an alternative clinical scale.

This pattern is consistent with previous adolescent research linking shorter sleep duration with depressive mood (39). Experimental evidence also suggests that restricted sleep may worsen negative mood and reduce energy among adolescents (40).

Several temporal explanations remain plausible. Short sleep may be associated with greater emotional vulnerability (41), while depressive symptoms may also precede or accompany sleep disruption and poorer perceived sleep quality (42). Exercise, sleep, and depressive symptoms may additionally be related to common underlying factors, including academic demands, screen use, school schedules, family routines, socioeconomic circumstances, physical health, and unmeasured stressors (43, 44). The present analyses did not quantify time-use substitution and therefore cannot determine whether exercise displaced sleep, study time, screen use, or other daily activities. Because all variables were measured in the same survey wave, the present study cannot determine the direction of these relationships.

### 4.4 Robustness and Age-group analyses

Results were generally similar using CES-D7 and the operational binary CES-D8 outcome. Multiple-imputation estimates for exercise participation, exercise-frequency categories, and the prespecified sleep-duration contrasts were also broadly consistent with the complete-case estimates, although the 9-hour vs. 8-hour contrast was less precise after imputation.

Additional adjustment for educational years, health indicators, and happiness attenuated the exercise estimates more than the sleep estimates. These variables were measured concurrently and may represent confounders, correlates, or consequences of exercise and depressive symptoms. The attenuation should therefore not be interpreted as mediation or as evidence that one covariate specification was uniquely correct. Instead, it indicates that the exercise associations were more sensitive to model specification and may partly reflect residual socioeconomic, health, or psychological differences.

No statistically detectable interactions with age group were observed for exercise frequency, exercise participation, participant session duration, or sleep duration. The available data therefore provided no strong evidence that these associations differed between adolescents aged 10–14 and 15–19 years. However, the absence of a statistically significant interaction does not demonstrate equivalence, particularly because estimates for the less common exercise-frequency categories were relatively imprecise.

The findings support considering exercise and sleep as related but distinct dimensions of adolescent health behavior. Schools, families, and public-health practitioners may continue to provide accessible opportunities for regular physical activity and to support adequate sleep opportunities. However, the data do not justify prescribing a particular exercise-frequency category or extending the duration of individual exercise sessions as a strategy for reducing depressive symptoms.

The nonmonotonic exercise-frequency pattern suggests that exercise quality and context may warrant greater attention in future program design and evaluation, including sustainability, enjoyment, safety, social inclusion, and compatibility with school and sleep schedules. Similarly, the sleep findings support attention to insufficient sleep, but they do not establish that increasing sleep duration would necessarily reduce depressive symptoms in every adolescent. Longitudinal and intervention studies are needed to determine whether changes in these behaviors precede changes in mental health.

### 4.5 Limitations

Several limitations should be acknowledged. First, the cross-sectional design precludes conclusions about temporal ordering or causality. Depressive symptoms may precede or accompany changes in exercise and sleep, and bidirectional associations are plausible. Second, exercise, sleep duration, and depressive symptoms were self-reported and were assessed over different reference periods, which may have introduced recall error and limited temporal alignment.

Third, the available exercise measures did not capture intensity, total weekly volume, activity type, timing, enjoyment, or competitive context. Sleep quality, insomnia symptoms, bedtime, wake time, and circadian timing were also unavailable. Potential confounders such as academic workload, screen and sedentary time, chronic health conditions, medication use, family structure, parental mental health, and school characteristics were unavailable, incompletely measured, or not harmonized across the full sample.

Fourth, complete-case restrictions and missing survey-design information may have affected representativeness. Multiple imputation addressed eligible missing covariates but could not account for missing CES-D8 scores, invalid design variables, invalid participant session duration, or data missing not at random. The 10–19-year age range spans substantially different developmental and school stages. Although exploratory interaction tests found no statistically detectable differences between adolescents aged 10–14 and 15–19 years, this two-group comparison may not capture finer heterogeneity in pubertal development, cognitive and emotional maturity, school context, daily routines, exercise behavior, or sleep needs.

Fifth, the study was conducted among adolescents in China, and the observed associations may not generalize directly to populations with different cultural, educational, family, and health-behavior contexts. Replication in other countries and sociocultural settings is needed. Finally, category-specific exercise and sleep contrasts were not adjusted for multiple comparisons. Estimates close to conventional significance thresholds, particularly the 9-hour sleep contrast, should therefore be interpreted cautiously.

## 5 Conclusion

Exercise frequency and sleep duration were modestly associated with depressive-symptom scores among Chinese adolescents, whereas session duration among exercise participants showed no clear association. The exercise-frequency pattern was nonmonotonic, and the sleep association was most evident at shorter durations. Longitudinal studies using repeated and objective measures are needed to clarify whether changes in exercise and sleep precede changes in adolescent depressive symptoms.

## Data Availability

The data analyzed in this study is subject to the following licenses/restrictions: the datasets used in this study (CFPS) are available from the official CFPS data platform upon successful application. In accordance with CFPS policy, these datasets are distributable only by the CFPS team. Requests to access these datasets should be directed to https://cfpsdata.pku.edu.cn/#/home.
